# Soluble Alpha-Klotho Alleviates Cardiac Fibrosis without Altering Cardiomyocytes Renewal

**DOI:** 10.3390/ijms21062186

**Published:** 2020-03-22

**Authors:** Wei-Yu Chen

**Affiliations:** Institute for Translational Research in Biomedicine, Kaohsiung Chang Gung Memorial Hospital, Kaohsiung City 83301, Taiwan; wychen624@cgmh.org.tw

**Keywords:** klotho, FGF-23, cardiac fibrosis, genetic fate mapping

## Abstract

Heart disease is the leading cause of death worldwide. The major cause of heart failure is the death of the myocardium caused by myocardial infarction, detrimental cardiac remodeling, and cardiac fibrosis occurring after the injury. This study aimed at discovering the role of the anti-aging protein α-klotho (KL), which is the co-receptor of fibroblast growth factor-23 (FGF23), in cardiac regeneration, fibrosis, and repair. We found that the anti-apoptotic function of soluble KL in isoproterenol-treated H9c2 cardiomyocytes was independent of FGF23 in vitro. In vivo, isoproterenol-induced cardiac fibrosis and cardiomyocyte and endothelial cell apoptosis were reduced by KL treatment. Moreover, the number of Ki67-positive endothelial cells and microvessel density within the isoproterenol-injured myocardium were increased upon KL treatment. However, by using genetic fate-mapping models, no evident cardiomyocyte proliferation within the injured myocardium was detected with or without KL treatment. Collectively, the cardioprotective functions of KL could be predominantly attributed to its anti-apoptotic and pro-survival activities on endothelial cells and cardiomyocytes. KL could be a potential cardioprotective therapeutic agent with anti-apoptotic and pro-survival activities on cardiomyocytes and endothelial cells.

## 1. Introduction

Congestive heart failure is a leading cause of morbidity and mortality worldwide. There are an estimated 4.9 million cases of heart failure in the United States alone. Currently, 80% of men and 70% of women die within 8 years of diagnosis (American Heart Association. Heart Disease and Stroke Statistics—2020 Update) [1]. The major non-surgical treatments for heart failure include administration of beta-blockers, angiotensin-converting-enzyme (ACE) inhibitors, aldosterone antagonists, and symptomatic treatment. However, these treatments are inadequate, and heart failure remains a progressive and often lethal disease in the majority of cases. Whereas the heart of some vertebrates like zebrafish and newts can undergo significant regeneration after injury, the myocardial regenerative response in adult mammals is dramatically reduced [2,3]. Although recent studies demonstrate that humans can generate new heart cells [4], regeneration in the adult human heart is grossly inadequate to compensate for the severe loss of cardiomyocytes following myocardial infarction. Thus, understanding how to guide heart cell regeneration is a critical goal.

The cellular origin of postnatal cardiomyogenesis in mammals remains a controversial issue. Two mechanisms seem to be participating—myogenic differentiation of progenitor cells and proliferation of pre-existing cardiomyocytes [5]. Although adult cardiomyocyte division may contribute to cardiac regeneration [6,7,8], cardiac progenitor cells can lead to new heart cells [9]. A growing body of evidence suggests that the adult mammalian heart preserves a self-renewal capacity, and stem/progenitor cell populations reside in it [10,11,12,13,14,15,16,17]. Additionally, approximately 20% of cardiomyocytes are replenished by endogenous stem/progenitor cells in the peri-infarct border zone after myocardial infarction (MI) in mice [18,19,20]. These results imply that the majority of replenished cardiomyocytes may originate from endogenous stem/progenitor cells. However, the relative importance of these two mechanisms remains controversial (reviewed in ref [10,21,22,23]), and both show promising therapeutic potential [24]. Nonetheless, stem cell-mediated regenerative therapies still hold great promise for patients suffering from heart failure. However, a deeper understanding of the signals that control progenitor cell fate is required to further advance these therapies.

Advances in mouse genetic engineering allow cell tracking using a “fate-mapping” approach. For example, Dor and colleagues designed a “pulse-chase” strategy to study pancreatic β-cell turnover in adult mice using an inducible Cre-loxP system [25]. This strategy for studying cardiomyocyte refreshment was published in a landmark study by Hsieh et al. [18] and has then been applied to study factors influencing cardiomyocyte regeneration and their role in stem cell therapy [18,19,26,27]. This fate-mapping approach relies on an inducible cardiomyocyte-specific transgenic mouse to demonstrate the refreshment of cardiomyocytes from stem/precursor cells. Hsieh et al. [18] generated double-transgenic MerCreMer/ZEG mice by crossbreeding a mouse strain in which a cardiomyocyte-specific α-myosin heavy chain promoter drives the expression of an engineered 4-OH-Tamoxifen-inducible Cre recombinase fusion protein (MerCreMer) [28] with the ZEG reporter strain, in which constitutive β-galactosidase expression is replaced by enhanced green fluorescence protein (GFP) expression upon the removal of a loxP-flanked stop sequence [29]. Therefore, following treatment of the MerCreMer/ZEG mice with 4-OH-Tamoxifen at the selected time interval (the “pulse”), cardiomyocytes become irreversibly labeled with GFP. During normal aging or following experimental myocardial infarction (MI) or pressure overload (the “chase”), the percentage of these GFP+ cardiomyocytes may remain constant or decrease. Stem/precursor cells, which do not express the α-myosin heavy chain gene, would not be genetically labeled with GFP. Thus, the percentage of GFP+ cardiomyocytes will decrease over time if stem/precursor cells replenish cardiomyocytes (dilution by GFP-negative cells), or it will remain unchanged if stem/precursor cells do not contribute to cardiomyocyte renewal or regeneration.

Using the same model, Loffredo et al. further demonstrated that transplantation of c-Kit+ bone marrow stem cells (BMSC) contributed to cardiomyocyte refreshment after MI [19]. In the c-Kit+ BMSC transplanted heart, the population of β-gal+ cells was increased, whereas that of the GFP+ cells was decreased, indicating that cell transplantation promoted cardiac refreshment [19]. A recent study by Hsueh et al., using the same model, indicated that the critical period for stem/progenitor cell-mediated cardiomyocyte replenishment was initiated within 7 days and saturated on day 10 post-infarction [27]. The same study also showed that cardiac stem cells were PGE2-responsive and that PGE2 might regulate stem cell activity directly through the EP2 receptor or indirectly by modulating the cell micro-environment in vivo [27]. Additionally, it provided evidence that PGE2 had the ability to lower the expression of transforming growth factor (TGF)-β1—which is an aging marker gene—and rejuvenate aged cells by altering their micro-environment. This observation holds great potential for cardiac regeneration and offers opportunities for developing new de novo treatments.

Fibroblast growth factor-23 (FGF23) is a hormone produced by osteoblasts/osteocytes that acts on the kidney to regulate phosphate and vitamin D metabolism through the activation of FGF receptor/α-klotho (KL) co-receptor complexes. In addition, elevated levels of circulating FGF23 have been associated with left ventricular hypertrophy (LVH), and it has been suggested that FGF23 directly affects the myocardium [30]. However, the detailed mechanism of how FGF23 is involved in the process of cardiac fibrosis, remodeling, and cardiomyocyte refreshment after myocardium injury is unclear. It is possible that the negative effects of elevated FGF23 may depend on mechanisms different from those regulating phosphate homeostasis.

In this study, we investigated the anti-aging protein KL on cardiac repair and regeneration by using mouse models. We found that the administration of soluble KL exerted a cardioprotective function, independently of FGF23, both in vitro and in a mouse model of isoproterenol-induced cardiac fibrosis. Based on Cre-LoxP-based genetic fate mapping models, we demonstrated that soluble KL mitigated cardiac fibrosis and prevented endothelial apoptosis without affecting cardiomyocyte renewal. In conclusion, treatment with soluble KL represented a potential treatment for alleviating cardiac fibrosis in mouse models.

## 2. Results

### 2.1. Klotho (KL) Attenuated Isoproterenol-Induced Cell Death in H9c2 Cardiomyocytes In Vitro

**Figure 1:**
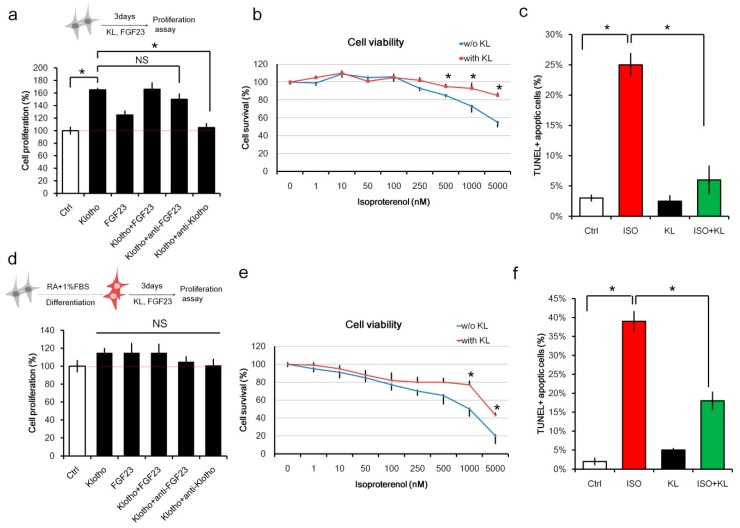
KL (α-klotho) attenuated isoproterenol-induced cell death in H9C2 cardiomyocytes. (**a**) Un-differentiated H9c2 cells were cultured with KL (200 ng/mL), fibroblast growth factor-23 (FGF23) (100 ng/mL), KL (200 ng/mL) plus FGF23 (100 ng/mL), KL (200 ng/mL) + anti-FGF23 (1 μg/mL), or KL (200 ng/mL) plus anti-KL (1 μg/mL) for 3 days. Cell proliferation activity was measured on day 3 using the Cell counting kit-8 (CCK-8) kit. (**b**) Un-differentiated H9c2 cells were cultured with different doses of isoproterenol in the presence or absence of KL (200 ng/mL). Cell viability was measured 48 h after the treatments using the CCK-8 kit. (**c**) TUNEL-positive cells were analyzed after immunofluorescence staining of cells treated with isoproterenol (1000 nM), KL (200 ng/mL), or isoproterenol (1000 nM) plus KL (200 ng/mL). Cells were fixed and stained 24 h post-treatment. (**d**) H9c2 cells were cultured in retinoic acid (RA) and 1% FBS for 5 days to induce differentiation and maturation. Differentiated H9c2 cells were then cultured with KL (200 ng/mL), FGF23 (100 ng/mL), KL (200 ng/mL) plus FGF23 (100 ng/mL), KL (200 ng/mL) + anti-FGF23 (1 μg/mL), or KL (200 ng/mL) plus anti-KL (1 μg/mL) for 3 days. Cell proliferation activity was measured on day 3 using the CCK-8 kit. (**e**) Differentiated H9c2 cells were cultured with different doses of isoproterenol in the presence or absence of KL (200 ng/mL). Cell viability was measured 48 h after the treatments using the CCK-8 kit. (**f**) TUNEL-positive cells were analyzed after immunofluorescence staining of cells treated with isoproterenol (1000 nM), KL (200 ng/mL), or isoproterenol (1000 nM) plus KL (200 ng/mL). Cells were fixed and stained 24 h post-treatment. In all graphs, * indicates *p* < 0.05. Data are representative of three independent experiments.

To investigate whether the FGF23/KL axis promoted cardiomyocyte proliferation, we analyzed the effect of FGF23 and KL on un-differentiated H9c2 cardiomyoblast cells. H9c2 cells were treated with FGF23, KL, or FGF23 plus KL for 3 days. Cell proliferation was analyzed using the CCK-8 kit. We found that KL alone, or KL in combination with FGF23, promoted cell proliferation (Figure 1a). Interestingly, anti-FGF23 did not affect KL-mediated cell proliferation, suggesting that FGF23 was not required for KL-mediated cell proliferation. To determine the effect of KL on isoproterenol-induced cell death, viability assays were performed on H9c2 cells treated with different concentrations of isoproterenol in the presence or absence of KL. KL significantly reduced isoproterenol-mediated cellular death (Figure 1b). TUNEL immunofluorescent staining also showed that KL treatment reduced isoproterenol-induced cell apoptosis (Figure 1c).

It has been shown that RA and low FBS promote H9c2 differentiation toward a mature cardiomyocyte phenotype [31]. Therefore, we also examined whether RA-differentiated H9c2 cells also respond to FGF23/KL treatment. Our results demonstrated that neither FGF23 nor KL affected cell proliferation in RA-differentiated H9c2 cells (Figure 1d). On the contrary, cell viability assays and TUNEL staining showed that KL inhibited isoproterenol-induced cell death (Figure 1e) and apoptosis (Figure 1f) in RA-differentiated H9c2 cardiomyocytes. These results indicated that KL protected against isoproterenol-induced cell death in both undifferentiated and differentiated H9c2 cells, whereas it promoted the proliferation of only undifferentiated H9c2 cells.

### 2.2. KL Inhibited Isoproterenol-Induced Cardiac Fibrosis and Cellular Apoptosis In Vivo

**Figure 2:**
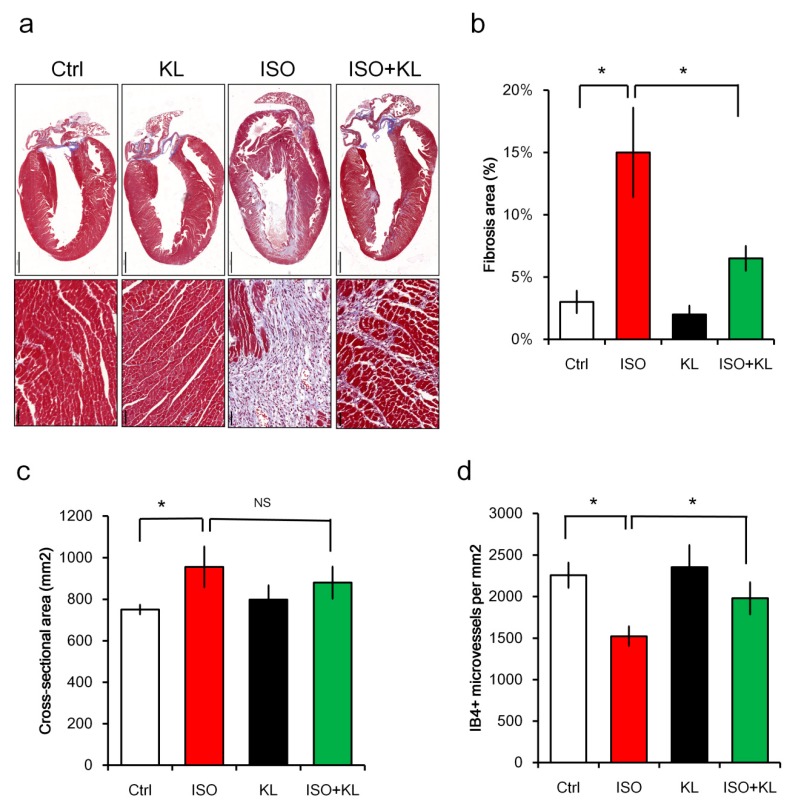
KL inhibited isoproterenol-induced cardiac damage in vivo. Balb/c mice (*n* = 10 in each group) were treated with saline control (Ctrl, normal saline in 100 μL, s.c.), isoproterenol (ISO) (60 mg/kg/day for 3 days, s.c.), KL (0.5 μg/mice/days for 5 days, i.p.), or ISO plus KL for 3 days. Mice were sacrificed, and their hearts were collected for Masson’s Trichrome staining for tissue fibrosis (**a**), and for measurements of fibrosis area (**b**), and cardiomyocyte cross-sectional area (**c**). (**d**) Quantification of isolectin B4-stained microvessels. * indicates *p* < 0.05. Bars represent 1000 μm and 50 μm in the upper and lower panel of Figure 2a, respectively.

We examined whether administration of soluble KL exerted a cardioprotective function in a mouse model of cardiac injury induced by isoproterenol. Balb/c mice (*n* = 10 in each group) were injected with saline control, isoproterenol, KL, or isoproterenol + KL for 3 days. Mice were sacrificed for histological assessments on day 5 after the last isoproterenol administration. We found that KL treatment inhibited isoproterenol-induced cardiac fibrosis using the Masson’s Trichrome staining (Figure 2a,b). However, we did not observe significant differences in the cardiomyocyte cross-sectional area between the isoproterenol and isoproterenol + KL groups (Figure 2c). We next examined whether KL affected the heart microvessel density following acute injury. Immunohistochemical staining of isolectin B4 (IB4) revealed that isoproterenol treatment caused the loss of myocardial endothelial cells, which was recovered by KL (Figure 2d).

### 2.3. KL Attenuated Isoproterenol-Induced Apoptosis of Cardiomyocytes and Endothelial Cells

**Figure 3:**
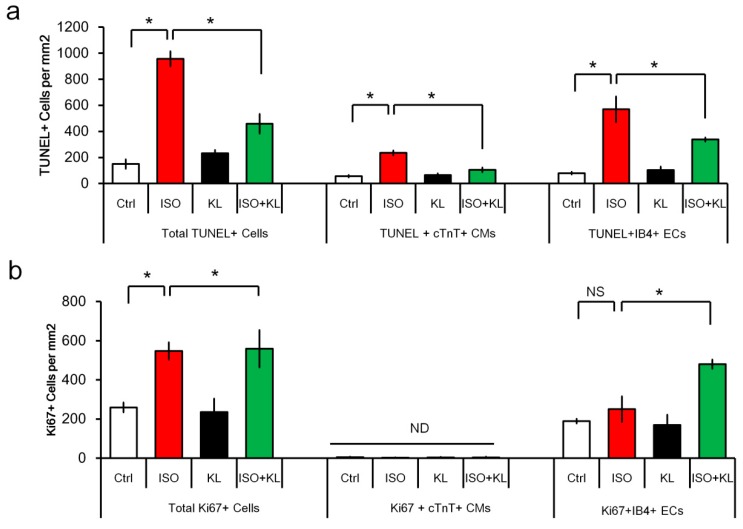
KL inhibited isoproterenol-induced cell death in vivo. (**a**) Quantification of immunofluorescent staining for TUNEL+ cells, TUNEL+cTnT+ cardiomyocytes, and TUNEL+IB4+ endothelial cells in the cardiac tissues. (**b**) Quantification of immunofluorescent staining for Ki67+ cells, Ki67+cTnT+ cardiomyocytes, and Ki67+IB4+ endothelial cells in the cardiac tissues. * denotes *p* < 0.05. NS, no significance. ND, not detectable.

We performed immunofluorescent staining to identify apoptotic cells in the cardiac tissues. The number of total TUNEL+ cells in the myocardium was increased following isoproterenol treatment, but the number of TUNEL+ cells was significantly reduced after KL treatment (Figure 3a). We next identified which cell type constituted the major population undergoing isoproterenol-induced apoptosis. Double TUNEL and cTnT or IB4 staining were performed to identify apoptotic cardiomyocytes (TUNEL+cTnT+) or endothelial cells (TUNEL+IB4+). Interestingly, the numbers of apoptotic cardiomyocytes and endothelial cells accounted for approximately 20 and 60% of the total apoptotic cells within the injured myocardium, respectively (Figure 3a). We found that KL reduced both the number of TUNEL+ apoptotic cardiomyocytes and endothelial cells (Figure 3a). These results indicated that KL exerted a cardioprotective function in a mouse model of cardiac injury through its anti-apoptotic and pro-survival activities.

### 2.4. KL Increased the Number of Proliferating Endothelial Cells but Not Cardiomyocytes

Isoproterenol caused an increased number of Ki67+ proliferating cells, whereas KL did not alter the total number of Ki67+ cells (Figure 3b). We performed double staining for the cell proliferation marker Ki67 and the cardiomyocyte marker cTnT to identify proliferating cardiomyocytes within the myocardium, and we found that the number of cTnT+ cardiomyocytes with Ki67-stained nuclei was extremely low (less than 10 cells could be identified per heart cross-section) (Figure 3b). IB4+ endothelial cells accounted for ~50% of the total Ki67+ cells. Interestingly, IB4+ endothelial cells accounted for ~80% of the total Ki67+ cells in the isoproterenol + KL group, which might imply that KL primarily increased the number of proliferating endothelial cells following cardiac injury (Figure 3b).

### 2.5. Generation of myh6-MerCreMer/GFP Reporter Mice for Studying Cardiomyocte Renewal

**Figure 4:**
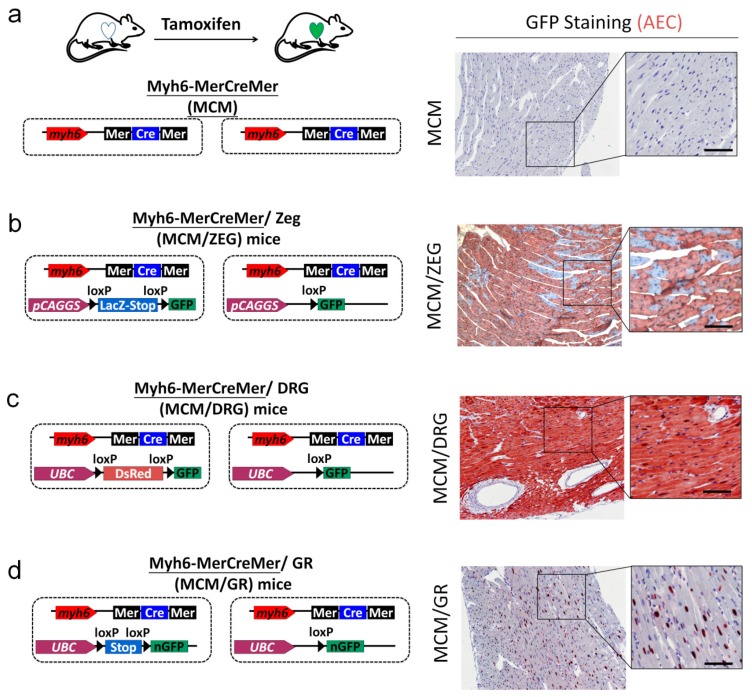
Generation of cardiomyocyte-specific reporter mouse models for genetic fate-mapping of adult cardiomyocytes. To establish a robust system for analyzing cardiomyocyte regeneration, we generated and compared the recombination efficiency of myh6-MerCreMer (MCM) mice with 3 different GFP reporter systems. GFP expression was detected by immunohistochemical staining in the cardiac tissues of MCM/reporter mice injected with Tamoxifen. (**a**) MCM mice without a GFP reporter were used as a negative control. (**b**) The myh6-MerCreMer strain was crossed with ZEG mice to create MCM/ZEG double heterozygous mice. Upon a Tamoxifen pulse, the reporter switches from β-galactosidase (encoded by *lacZ*) to cytosolic GFP expression in cardiomyocytes only. (**c**) The myh6-MerCreMer strain was crossed with DRG mice to create MCM/DRG double heterozygous mice. Upon Tamoxifen’s pulse, the reporter switches from DsRed to cytosolic GFP expression in cardiomyocytes only. (**d**) The myh6-MerCreMer strain was crossed with GR mice to create MCM/GR double heterozygous mice. Upon a Tamoxifen pulse, the reporter switches from non-GFP expression to nuclear GFP expression in cardiomyocytes only. Bars represent 50 μm.

To study cardiac regeneration by cardiomyocyte renewal in vivo, we established and optimized an in vivo assay platform based on three different cardiomyocyte-specific labeled GFP reporter mice. Here, we used the inducible cardiomyocyte-specific transgenic mouse fate-mapping approach to determine the frequency with which cardiomyocytes are refreshed from stem or precursor cells [17]. We generated double-transgenic MerCreMer-ZEG (MCM/ZEG) mice by crossbreeding transgenic B6129-Tg(Myh6-cre/Esr1)1Jmk/J (hereafter referred to as MerCreMer) mice (Figure 4a), in which the cardiomyocyte-specific α-myosin heavy chain (Myh6) promoter drives the expression of a Tamoxifen-inducible Cre recombinase fusion protein [17], with B6.Cg-Tg(ACTB-Bgeo/GFP)21Lbe/J (hereafter referred to as ZEG) reporter mice (Figure 4b), in which constitutive β-galactosidase expression is replaced by the expression of GFP upon the removal of a loxP-flanked stop sequence [17]. Cardiomyocytes in these MerCreMer-ZEG mice were irreversibly labeled with GFP following treatment with Tamoxifen at a selected time point (the ‘pulse’) (Figure 4b).

We also crossed the myh6-MerCreMer mice to DRG mice to create MCM/DRG mice, in which constitutive DsRed expression was replaced by the expression of cytosolic GFP upon the removal of a loxP-flanked stop sequence (Figure 4c). Finally, the myh6-MerCreMer mice were crossed to R26R-GR mice to create MCM/GR mice, in which the expression of nuclear GFP was turned on upon the removal of a loxP-flanked stop sequence after Tamoxifen injection (Figure 4d).

**Figure 5:**
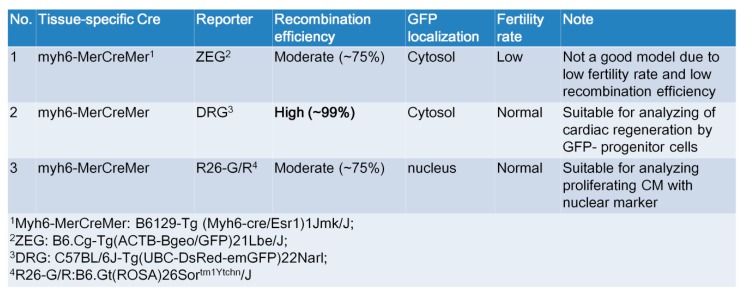
Comparison of the three GFP reporter mice model. The MCM/ZEG mouse model might not be a good model due to the low fertility rate and relatively low recombination efficiency. We identified the MCM/DRG as a better model to replace the MCM/ZEG model, and the MCM/GR as a suitable model for analyzing proliferating cardiomyocytes with nuclear markers.

We first examined the efficiency of cardiomyocyte labeling with GFP in 12-week-old MerCreMer-GFP reporter mice after Tamoxifen injection, as the experimental strategy depends upon high efficiency of GFP labeling in cardiomyocytes combined with little GFP labeling efficiency in non-cardiomyocytes. Following the Tamoxifen pulse, GFP expression was dramatically increased (Figure 4b–d). Following 10 days of Tamoxifen treatment, we quantified the percentages of GFP+ cardiomyocytes by using an automated color separation technique and found that the labeling efficiency in cardiomyocytes reached 75 ± 9.2%, 98 ± 2.3%, and 76 ± 6.3% levels in MCM/ZEG, MCM/DRG, and MCM/GR mice, respectively (Figure 4 and Figure 5). This result indicated that we established and validated the MCM/DRG model as an optimal model for studying cardiomyocyte renewal in vivo. Additionally, MCM/GR mice could also be used as a suitable model for the detection of proliferation or apoptotic markers in the nucleus of cardiomyocytes.

### 2.6. KL Ameliorated Isoproterenol-Induced Cardiac Fibrosis, Whereas Its Did Not Affect Cardiac Renewal in Adult Mice

**Figure 6:**
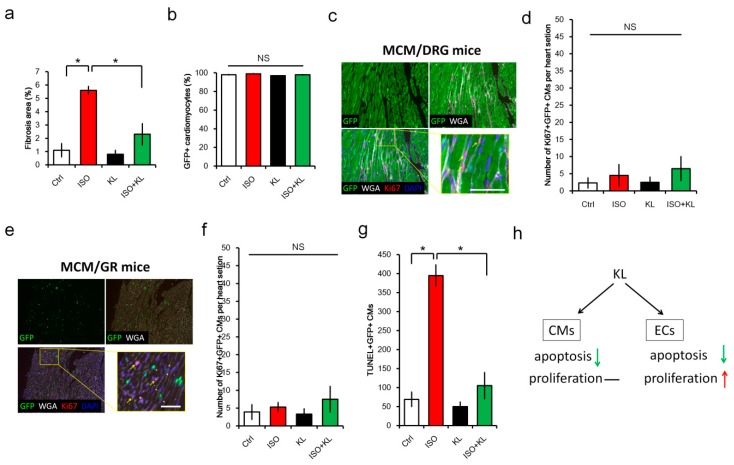
KL attenuated isoproterenol-induced cardiac fibrosis and cardiomyocyte apoptosis without altering cardiomyocyte renewal in adult mice. (**a**) MCM/DRG mice (*n* = 5 in each group) with isoproterenol-induced cardiac injury (100 mg/kg/day for 5 days) were injected with saline or KL recombinant protein. Cardiac fibrosis was analyzed on day 7 post-cardiac injury. (**b**) The percentage of GFP+ cardiomyocytes among cardiac troponin T (cTnT)+ cells was quantified using immunohistochemistry staining with a GFP antibody. (**c**) Localization of Ki67-positive non-myocardial cells in the heart of isoproterenol-treated MCM/DRG mice. Bars represent 50 μm. (**d**) The number of Ki67+GFP+ cardiomyocytes within the myocardium. (**e**) MCM/GR mice (*n* = 5 in each group) with isoproterenol-induced cardiac injury (100 mg/kg/day for 5 days) were injected with saline or KL recombinant protein. Localization of Ki67-positive non-myocardial cells in the heart from ISO-treated MCM/GR mice. Bars represent 50 μm. (**f**) The number of Ki67+GFP+ cardiomyocytes within the myocardium. (**g**) The number of TUNEL+GFP+ cardiomyocytes within the myocardium. (**h**) KL mediated its cardioprotective effect by inhibiting apoptosis and promoting the proliferation of endothelial cells following cardiac injury. No evident cardiomyocyte proliferation was found following isoproterenol-induced cardiac injury in the presence or absence of exogenous KL. NS denotes no significant differences, and * denotes *p* < 0.05.

To confirm whether recombinant KL protein could reduce stress-induced cardiac fibrosis and promote cardiac regeneration via cardiomyocyte renewal, we analyzed the cardioprotective effect of KL in MCM/DRG mice. We found that the administration of KL reduced cardiac fibrosis induced by isoproterenol in MCM/DRG mice (Figure 6a). To further examine whether the cardiac protective effect of KL was attributed to improved cardiac renewal by stem cells, we compared the percentage of GFP+ cardiomyocytes in heart tissues from each group. However, we did not observe significant differences in cardiomyocyte refreshment in this model (Figure 6b). We also analyzed the localization of Ki67-labeled proliferating cells and found that Ki67+ cells were mainly interstitial non-myocytes (Figure 6c). Only trace amounts (less than 10 cells per heart section) of Ki67+ GFP+ cardiomyocytes were detected among the groups, suggesting that the proliferation of cardiomyocytes minimally contributed to the KL-mediated cardioprotective function.

To further confirm this finding, we performed isoproterenol-induced cardiac injury in MCM/GR mice, which is a more suitable model to identify proliferating cardiomyocytes (nuclear Ki67 expression) with nuclear GFP expression (Figure 4d). However, only a few (less than 10 cells per heart section) Ki67-labeled cardiomyocytes with nuclear GFP expression were identified in MCM/GR mice (Figure 6e,f). Despite the lack of evidence for cardiomyocyte proliferation, we observed that the number of TUNEL+ apoptotic cardiomyocytes was significantly reduced by KL within isoproterenol-injured hearts (Figure 6g). Taken together, our results suggested that KL was cardioprotective, but its beneficial effects might not be attributed to the promotion of cardiomyocyte proliferation and cardiac regeneration in vivo. Instead, our results demonstrated that the KL-mediated cardioprotective function might be attributed to its anti-apoptotic effect on cardiomyocytes and endothelial cells (Figure 6h). Thus, KL might also selectively induce a pro-survival signal in cardiac endothelial cells within the injured myocardium.

## 3. Discussion

FGF23 is the most recently discovered FGF and functions as an endocrine hormone that regulates phosphate homeostasis through binding to FGFR and KL [32], its coreceptor in the kidney and parathyroid glands [33,34]. Elevated levels of circulating FGF23 have been associated with left ventricular hypertrophy, and it has been suggested that FGF23 exerts a direct effect on the myocardium [30]. Interestingly, the co-receptor of FGF23, KL, has been shown to exhibit renal protective functions independent of FGF23/FGFR signaling [35,36]. In this study, we identified KL as a novel cardioprotective factor with anti-apoptotic and pro-survival functions in cardiomyocytes and endothelial cells.

Soluble KL has recently been shown to regulate transient receptor potential canonical isoform 6 (TRPC6)-mediated calcium levels, independent of FGF23/FGFR receptor signaling [37]. In a mouse model of cardiac injury, KL has been shown to ameliorate isoproterenol-induced cardiac injury via reduction of oxidative stress [38]. In a mouse model of uremic cardiomyopathy, exogenous administration of soluble KL also exerts a beneficial effect independent of FGF23 and phosphate [35]. Taken together, these observations suggest that exogenous administration of soluble KL could be a potential treatment against cardiac fibrosis and that unidentified molecular mechanisms underlie its cardioprotective roles.

In this study, we generated and validated three Myh6-MerCreMer/GFP mice models for studying cardiomyocyte renewal: 1) MCM/ZEG, 2) MCM/DRG, and 3) MCM/GR. Although MCM/ZEG mice have been previously used for studying cardiomyocyte renewal in vivo [18], we found that the fertility rate of this line was very low (~2 to 3 mice per litter) in our hand. We also observed that Tamoxifen injection caused lethality in ~40% of MCM/ZEG mice, which was not described in the previous report. Thus, we considered that the maintenance of MCM/ZEG mice was not timely and cost-effective. Interestingly, we observed higher Cre recombination efficiency (~99%) and a higher fertility rate (~7–8 mice per litter) in MCM/DRG mice. Furthermore, no tamoxifen-induced lethality was observed in both MCM/DRG and MCM/GR mouse strains. Thus, we considered MCM/DRG mice as a more suitable in vivo model for studying cardiomyocyte renewal than MCM/ZEG mice. We also exploited the characteristic nuclear GFP expression in cardiomyocytes in MCM/GR mice, which might facilitate the co-localization of cell proliferating markers (such as Ki67 or PH3) with nuclear GFP in cardiomyocytes. Our results demonstrated the successful generation of genetic fate-mapping mouse models, which could serve as useful tools for the evaluation of cardiomyocyte renewal or the more precise determination of the expression of nuclear markers in cardiomyocytes in vivo (Figure 4 and Figure 5).

Our in vitro assays showed that KL alone promoted H9c2 cardiomyocyte proliferation and attenuated isoproterenol-induced cell apoptosis independent of the FGF23 pathway, suggesting that another unidentified mechanism underlay these soluble KL-mediated functions. In vivo, the administration of KL reduced isoproterenol-cardiac fibrosis and prevented cardiomyocyte and endothelial cell apoptosis. Although we did not observe a significant effect of KL in promoting cardiac regeneration in vivo, our animal model still provided a valuable assay platform for screening factors that might promote cardiac regeneration. Additionally, one limitation of our current study was that we did not perform cardiac functional assessments in our animal study. Further study is required to investigate whether KL could improve cardiac function in vivo. Collectively, the cardioprotective functions of KL were predominantly attributed to its anti-apoptosis and pro-survival activities on endothelial cells and cardiomyocytes. KL could be a potential cardioprotective therapeutic agent with anti-apoptotic and pro-survival activities on cardiomyocytes and endothelial cells.

## 4. Materials and Methods

### 4.1. Mice

Double transgenic MerCreMer-ZEG mice were generated by crossbreeding transgenic B6129-Tg (Myh6-cre/Esr1)1Jmk/J (MerCreMer) mice with the B6.Cg-Tg(ACTB-Bgeo/GFP)21Lbe/J reporter strain (ZEG, Jackson Laboratories), as previously described [18]. MerCreMer-DRG mice were generated by crossbreeding transgenic MerCreMer mice with the C57BL/6-Tg(UBC-flDsRed-emGFP)22Narl strain (RMRC13119, GEMMS, Taiwan). MerCreMer-GR mice were generated by crossbreeding transgenic MerCreMer mice with R26-G/R: B6-Gt(ROSA)26Sor^tm1Ytchn^/J mice (Stock No. 021847, kindly provided by Dr. Shu-Wha Lin, National Taiwan University) [39]. Colony maintenance and all experiments were conducted in accordance with the Guide for the Use and Care of Laboratory Animals and approved by the IACUC Committee (IACUC number: 2014031903) of Kaohsiung Chang Gung Memorial Hospital, Kaohsiung, Taiwan. All mice were maintained in the AAALAC certified animal facility in Kaohsiung Chang Gung Memorial Hospital.

### 4.2. Tamoxifen Pulse

A previously validated protocol was used to induce Cre recombinase-mediated GFP expression and permanent deletion of the β-galactosidase gene in cardiomyocytes of MerCreMer-GFP mice [18]. Tamoxifen (Sigma-Aldrich, St. Louis, MO, USA) was dissolved in peanut oil (Sigma) at a concentration of 5 mg/mL and injected intraperitoneally (i.p.) at a dose of 20 mg/kg/d for 5 days. Cre recombination in the cardiac tissue was analyzed by using immunochemistry to assess the ratio of GFP+ cardiomyocytes in the heart of the targeted mice.

### 4.3. Isoproterenol-Induced Cardiac Injury

To induce acute cardiac injury in male Balb/cByJ mice (National Laboratory Animal Center, Taipei, Taiwan), we injected isoproterenol (60 mg/kg/day) subcutaneously for 3 days. Mice were sacrificed on day 5 after the final isoproterenol injection. α-klotho (KL, 0.5 μg/mice/days) was injected i.p. immediately after the isoproterenol injection for 3 days. For the isoproterenol-induced cardiac injury in MCM/DRG and MCM/GR mice (C57BL/6 background), isoproterenol (100 mg/kg/day) was injected i.p. for 5 days with or without KL (0.5 μg/mice/days). Control mice were subcutaneously injected with normal saline. Mice were sacrificed on day 5 after the final isoproterenol injection.

### 4.4. Immunofluorescence Staining

Mouse hearts were fixed with 4% paraformaldehyde, paraffin-embedded, sectioned, stained, and imaged, following standard immunohistochemistry and fluorescent microscopy methods, as previously described [40,41]. An extra antigen retrieval step was added in all experiments by heating samples in a Tris-based buffer (pH 9) to 95 °C for 20 min. Primary antibodies against cardiac troponin T (cTnT) (AB8295, Abcam), Ki67 (AB16667, Abcam), GFP (SC-9996, Santa Cruz), FGF23 (SC-16849, Santa Cruz), Klotho (SC-22220, Santa Cruz), and Isolectin B4-biotin (I21414, Invitrogen) were used. Alexa Fluor conjugated secondary antibodies were purchased from ThermoFisher, Waltham, MA, USA). TUNEL staining was performed using an Apoptosis/Necrosis detection kit (Roche, Basel, Switzerland). Before mounting, the slides were incubated with 1% Sudan black (Sigma-Aldrich) in 75% ethanol at room temperature for 20 min to reduce tissue auto-fluorescence. Confocal images were acquired with an Olympus FLUOVIEW FV10i confocal microscope. Whole heart tissue section scanning was performed using TissueFAXS (TissueGnostics, Vienna, Austria). High magnification images of 10 fields of the cardiac tissue were randomly acquired with a light microscope (Olympus BX53). The number of Ki67+ cells or TUNEL+ cells in each image was determined with the Image J software (NIH, Bethesda, MD, USA).

### 4.5. Cell Viability Assay

Cell viability assays were performed with un-differentiated cardiomyocyte H9c2 cell lines, obtained from the America Tissue Type Collection (Manassas, VA, USA; catalog # CRL-1446). The cells were cultured in DMEM, supplemented with 1.5 g/L sodium bicarbonate, 10% fetal bovine serum (FBS), 100 U/mL penicillin, and 100 μg/mL streptomycin, in 10-cm tissue culture dishes at 37 °C in a humidified atmosphere of 5% CO_2_. The cell culture medium was replaced every 2–3 days and sub-cultured when reaching 70–80% confluence in order to prevent the loss of their differentiation potential. For cardiac cell differentiation, H9c2 cells were plated at a density of 35,000 cells/mL under cell differentiation conditions (1% FBS + retinoic acid (RA)) in 10-cm tissue culture dishes and cultured for one more day in high serum to allow for cell attachment. The addition of RA (1 μM) to media containing 1% serum was performed daily in the dark for 3 days. All-trans-RA was prepared in DMSO and stored at −20 °C in the dark to avoid degradation. To analyze the effect of KL on the proliferation of H9c2 cells, the cells were cultured with KL (200 ng/mL, R&D Systems #1819-KL-050), FGF23 (100 ng/mL, R&D Systems 2629-FG-025), KL (200 ng/mL) plus FGF23 (100 ng/mL), KL (200 ng/mL) + anti-FGF23 (1 μg/mL, Santa Cruz #sc-16849), or KL (200 ng/mL) plus anti-KL (1 μg/mL, Santa Cruz #sc-22220) for 3 days. Cell proliferation activity was measured on day 3 using the Cell counting 8 (CCK-8) kit (Sigma, FL-96992). The effect of KL on isoproterenol-induced cell death was assayed after treating cells with KL (200 ng/mL) in the presence of isoproterenol (1 nM, 10 nM, 50 nM, 100 nM, 250 nM, 500 nM, 1000 nM, 5000 nM). Cell survival was assessed using the CCK-8 kit, according to manufacturer’s instructions.

### 4.6. Statistical Analysis

Statistical significance between two groups was analyzed using the nonparametric Mann–Whitney test. The significance of differences among the groups was evaluated using a one-way analysis of variance, followed by the Bonferroni multiple comparison posthoc test. Statistical analysis was performed using Prism 6 statistical software (GraphPad, San Diego, CA, USA). All data are reported as the means ± SD. Statistical significance was set at *p* < 0.05.
